# Regulating Early Biological Events in Human Amniotic Epithelial Stem Cells Using Natural Bioactive Compounds: Extendable Multidirectional Research Avenues

**DOI:** 10.3389/fcell.2022.865810

**Published:** 2022-04-01

**Authors:** Farhana Ferdousi, Hiroko Isoda

**Affiliations:** ^1^ Alliance for Research on the Mediterranean and North Africa (ARENA), University of Tsukuba, Tsukuba, Japan; ^2^ Faculty of Life and Environmental Sciences, University of Tsukuba, Tsukuba, Japan; ^3^ AIST-University of Tsukuba Open Innovation Laboratory for Food and Medicinal Resource Engineering (FoodMed-OIL), AIST, University of Tsukuba, Tsukuba, Japan; ^4^ R&D Center for Tailor-made QOL, University of Tsukuba, Tsukuba, Japan

**Keywords:** human amniotic epithelial cells, natural compound, differentiation inducer, drug screening, cell priming, functional foods, biobank

## Abstract

Stem cells isolated from perinatal tissue sources possess tremendous potential for biomedical and clinical applications. On the other hand, emerging data have demonstrated that bioactive natural compounds regulate numerous cellular and biochemical functions in stem cells and promote cell migration, proliferation, and attachment, resulting in maintaining stem cell proliferation or inducing controlled differentiation. In our previous studies, we have reported for the first time that various natural compounds could induce targeted differentiation of hAESCs in a lineage-specific manner by modulating early biological and molecular events and enhance the therapeutic potential of hAESCs through modulating molecular signaling. In this perspective, we will discuss the advantages of using naturally occurring active compounds in hAESCs and their potential implications for biological research and clinical applications.

## Graphical Abstract

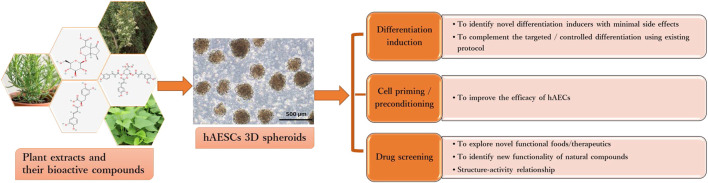

## Introduction

The term placenta is considered as an exploitable source of a number of pluripotent stem cells including, human amniotic epithelial stem cells (hAESCs), human amniotic mesenchymal stromal cells (hAMSCs), and human umbilical cord mesenchymal stromal cells (hUMSCs) (Miki and Strom, 2006; Ilancheran et al., 2007; Toda et al., 2007; Hu et al., 2009; Antoniadou and David, 2016; De Coppi and Atala, 2019). As derived from the biological waste product placenta, these perinatal stem cells are readily available, have an abundant supply, require no invasive harvesting procedures as well as have minimal ethical constraints. However, hAESCs possess unique biological characteristics compared to other perinatal pluripotent cells because of their developmental origin from the epiblast at around eight days after fertilization (Miki et al., 2005). They are derived from the innermost single layer of epithelial cells of the amnion that contacts the amniotic fluid directly. Isolated hAESCs express octamer-binding transcription factor-4 (OCT-4), a key transcription factor that maintain pluripotency and self-renewal in embryonic stem cells (ESCs) and induced pluripotent stem cells (iPSCs). hAESCs also express other pluripotent stem cell markers, such as Nanog homeobox (NANOG), SRY-Box transcription factor 2 (SOX2), stage-specific embryonic antigen (SSEA)3 and SSEA4, and tumor rejection antigen (TRA)1-60 and TRA 1-80 (Miki et al., 2005; Miki et al., 2010; Murphy et al., 2010; Gaggi et al., 2020). hAESCs lack telomerase activity and have short telomeres, which limit their proliferation efficiency (Gaggi et al., 2020). However, because of their limited proliferation capacity, hAESCs do not pose the risk of tumor or teratoma formation like ESCs (Miki et al., 2005). Moreover, under appropriate differentiation protocol, hAESCs can be differentiated into cells from all three germ layers, such as cells from the endodermal origin-liver, pancreas and lung epithelium, neural cells from the ectodermal origin, and bone and fat cells from mesodermal origin (Sakuragawa et al., 1996; Cai et al., 2005; Miki et al., 2005; Pan et al., 2006; Toda et al., 2007; Miki et al., 2010; Niknejad et al., 2010; Serra et al., 2018; Furuya et al., 2019). Notably, hAESCs have distinct expression profiles of human leukocyte antigens (HLAs). hAESCs show low expression of classical HLA-I: HLA-A, B, and C and no expression of HLA-II: HLA-DP, DQ, and DR, which contribute to immune recognition and rejection of PSCs after transplantation. hAESCs also express non-classical HLA-I: HLA-E, F, and G, specifically HLA-G, which have inhibitory effects on immune cells (Akle et al., 1981; Li et al., 2005). Thus, hAESCs are regarded as a promising source of stem cells in biological research and regenerative medicine.

On the other hand, natural resource-derived biologically active compounds, such as polyphenols, flavonoids, tannins, terpenoids, and fatty acids, have long been investigated for promoting cell division, and differentiation of pluripotent and adult stem cells (PSCs) under standard culture conditions (Udalamaththa et al., 2016; Udagama and Udalamaththa, 2018). Effects of plant extracts and their bioactive compounds on the proliferation and differentiation of mesenchymal stem cells (MSCs) have been extensively studied (Kornicka et al., 2017; Saud et al., 2019; Maeda, 2020). However, in spite of the fact that hAESCs were discovered nearly two decades ago, only a few studies have attempted to investigate the effects of natural compounds in hAESCs. As part of our continual effort to explore the bioactivities and functionalities of natural compounds of plant origin, we have been investigating their effects on modulating the early biological events in hAESCs (Ferdousi et al., 2019; Aonuma et al., 2020; Ferdousi et al., 2020; Uchida et al., 2020; Bejaoui et al., 2021; Ferdousi et al., 2021; Takahashi et al., 2021). In this perspective, we will discuss the multidirectional research opportunities through integrating natural bioactive compounds with the existing hAESCs research platforms.

## Natural Compound-Treated hAESCs: Potential Research Opportunities

### Natural Bioactive Compounds as Promising Differentiation Inducers of hAESCs

As hAESCs are derived from the pluripotent epiblast, these cells exert a high level of differentiation plasticity. A series of studies demonstrated successful induction of hAESCs into hepatocyte-like cells (Marongiu et al., 2011; Maymó et al., 2018; Furuya et al., 2019), hepatic sinusoidal endothelial cells (Serra et al., 2018), insulin-producing pancreatic *β* cells (Szukiewicz et al., 2010) through a combined approach using growth factors, cytokines, extracellular matrix proteins, or cocultured with mouse hepatocytes. Similarly, following treatment with noggin, serum, basic fibroblast growth factor (bFGF), and retinoic acid, hAESCs are able to differentiate into neural cells (Ishii et al., 1999; Okawa et al., 2001; Niknejad et al., 2010). Additionally, proper culture condition also induces mesodermal-lineage cells, including adipocytes, osteocytes, chondrocytes, and cardiomyocytes (Miki and Strom, 2006; Fang et al., 2012). Therefore, hAESCs provide an excellent cell source for cell therapy and regenerative medicine. However, hAESCs consist of a heterogeneous cell population according to different stem cell markers profiling (Centurione et al., 2018), which hinders the large-scale clinical transformation of hAESCs.

Additionally, the recombinant growth factors, synthetic and semi-biological cytokines, and proteins used for maintaining proliferation and inducing differentiation of stem cells, are reported to have toxic effects and possible risk of rejection. Also, these reagents are rapidly degradable and are required to replace continuously, making the whole procedure highly expensive, hence limiting their use in therapeutic tissue engineering (Marion and Mao, 2006; Raghavan et al., 2013). In this regard, exploring new biological approaches to facilitate hAESCs differentiation potential is highly needed.

**FIGURE 1:**
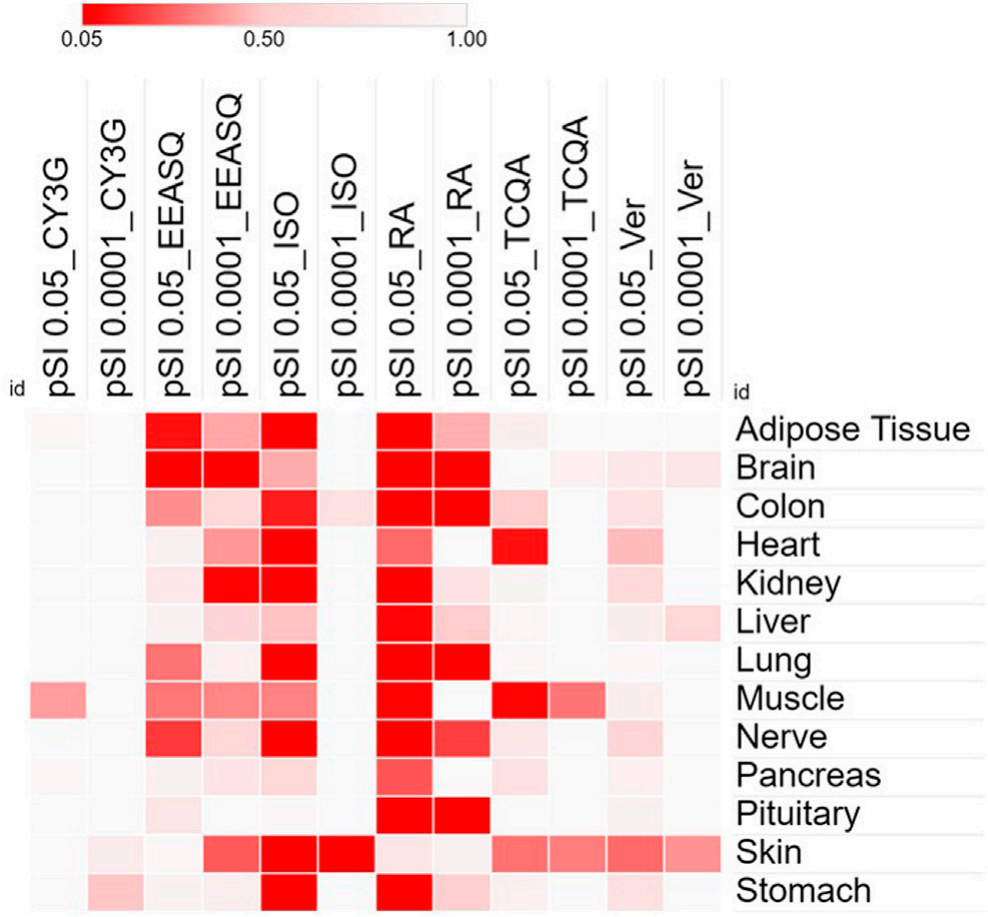
Heat map showing the significance and specificity of the tissue expressions by the differentially expressed genes of different compounds in hAESCs. Cells were treated with compounds for 7–10 days and RNAs were isolated from the control and treated hAESCs for microarray experiments using the Affymetrix’s GeneAtlas^®^ System (Affymetrix Inc., Santa Clara, CA, USA, human genome array strips; HG-U219). Genes with a linear fold change >1.1 (verbenalin), 1.2 (RA, TCQA and EEASQ), and 2 (ISO, CY3G) and a *p*-value < 0.05 (one-way between-subjects ANOVA) were considered as differentially expressed genes. Enrichment analysis was conducted using the Tissue Specific Expression Analysis (TSEA) tool (http://genetics.wustl.edu/jdlab/tsea//). Heat map was generated on Morpheus tool (https://software.broadinstitute.org/morpheus//). Significance of tissue enrichment were identified by Fisher’s Exact test. pSI, Specificity Index thresholds; pSI 0.05, significantly enriched all transcripts; pSI 0.0001, most specific subset of significantly enriched transcripts; CY3G, cyanidin 3-glucoside; EEASQ, ethanol extract of Aurantiochytrium-derived squalene; ISO, isorhamnetin; RA, rosmarinic acid; TCQA, 3,4,5-tri-O-caffeoylquinic acid; Ver, verbenalin.

In recent years, a new research stream has been developing to use naturally occurring bioactive compounds as stimulants of stem cells because of their high availability, low toxicity, and minimum side effects. Certain phytochemicals have been extensively studied for adult stem cell proliferation and inhibition of cancer cell proliferation (Udalamaththa et al., 2016). Those plant-derived pharmacologically active substances are reported to increase the rate of cell division and differentiation through modulating complex signal pathways and to facilitate tissue regeneration and immunomodulation. However, in hAESCs, the effects of natural compounds have not been explored widely. In our previous studies, we have reported for the first time that several natural compounds could regulate early biological events in hAESCs suitable for controlled differentiation of hAESCs. A caffeic acid ester, rosmarinic acid (RA), showed the potential of enhanced neural cell differentiation in hAESCs through downregulating the gene expressions related to canonical WNT pathway, BMP/TGF-b pathway, and notch signaling pathway (Ferdousi et al., 2019). RA also upregulated the expression of nemo like kinase (*NLK*), the positive effector of non-canonical WNT pathway. A caffeoylquinic acid derivative, 3,4,5-Tri-O-Caffeoylquinic acid (TCQA), enhanced the expressions of catenin beta 1 (*CTNNB1*), bone morphogenetic protein 5 (*BMP5*), versican (*VCAN*), melanocortin 1 receptor (*MC1R*), and dermokine (*DMKN*) in hAESCs, which are known to be involved in neural and pigment cell differentiation (Bejaoui et al., 2021). A flavonol aglycone isorhamnetin could induce the expression of several hepatic progenitor markers, like delta-like non-canonical Notch ligand 1 (*DLK1*), epithelial cell adhesion molecule (*EPCAM*), and albumin (*ALB*). Isorhamnetin-treated hAESCs also showed several mature hepatocyte functions, including ICG uptake, glycogen storage, and urea production, and weak hepatic cytochrome P450 (CYP) enzyme activity (Uchida et al., 2020). An anthocyanin, cyanidin 3-glucoside (CY3G), upregulated the expression of meteorin like glial cell differentiation regulator (*METRNL*) in hAESCs, which is an adipomyokine with pleiotropic activities in adipose tissue (Takahashi et al., 2021). These findings in hAESCs are supported by previous studies on these compounds in different *in vitro* and *in vivo* settings. For example, RA has been reported to exert neuroprotective effects in neuroinflammatory and neurodegenerative diseases (Takeda et al., 2002; Ito et al., 2008; Sasaki et al., 2013; Kondo et al., 2015; Makhathini et al., 2018), which has been attributed to RA’s capacity to induce neural differentiation and neurotransmitter release. Similarly, TCQA has been reported to improve cognitive function in aging model mice through inducing adult neurogenesis (Sasaki et al., 2019a). TCQA has also been reported to promote hair regrowth and pigmentation *in vitro* and *in vivo* (Bejaoui et al., 2019; 2020). Isorhamnetin has been widely reported to alleviate hepatic fibrosis in a number of *in vivo* models (Lee et al., 2008; Ganbold et al., 2019; Liu et al., 2019), while CY3G is known for its anti-obesity and anti-diabetic effects through modulating adipocyte differentiation (Matsukawa et al., 2015; Olivas-Aguirre et al., 2016; Saulite et al., 2019). In Figure 1, we have shown the enriched cell types by differentially expressed genes in different compound-treated hAESCs. Detailed experimental and analysis procedures are available in our previously published paper (Ferdousi et al., 2019). In the future, establishing the optimal hAESCs culture procedure by utilizing appropriate preconditioning with natural compounds is worth further investigation.

### Natural Bioactive Compounds to Enhance Therapeutic Potential of hAESCs

The distinct immunomodulatory properties of hAESC make it the most promising candidate for cell-based therapy (Miki, 2011). Specifically, hAESCs have very low immunogenicity, thus are suitable for allotransplantation. Indeed, mounting studies have revealed the beneficial outcomes of hAESCs-based therapy for wound healing (Zhao et al., 2018; Zheng et al., 2018), skin graft (Li et al., 2012), injury repair (Kamiya et al., 2005; Parmar et al., 2006; Bai et al., 2020), pulmonary and liver fibrosis (Manuelpillai et al., 2012; Tan et al., 2014; Miki, 2016; Tan et al., 2017; Cargnoni et al., 2018), and importantly in neurological diseases (Di Germanio et al., 2016; Sanluis-Verdes et al., 2017), including spinal cord injury (Gao et al., 2014), Parkinson’s disease (Yang et al., 2010), Alzheimer’s disease (AD) (Xue et al., 2012; Kim et al., 2020), and multiple sclerosis (McDonald et al., 2011; Liu et al., 2012). However, successful clinical outcomes of hAESC transplantation depend on its immunomodulating functions. A previous study showed that expansion of hAESCs in serum-free culture media leads to significantly different expressions of stem cell markers, increased differentiation capacity and immunosuppression (Yang et al., 2018). Another study reported that prolonged exposure of hAESCs to the inflammatory cytokines, namely interleukin (IL)-1β and interferon (INF)-*γ*, resulted in enhanced secretion of immunomodulatory molecules (Kolanko et al., 2019). However, while current studies focus on the safety and efficacy of translating hAESC-based therapy into clinical practices, using natural compounds for priming approaches to improve the therapeutic efficacy of hAESCs has not been explored.

Our previous studies showed that treatment with natural compounds increases anti-inflammatory potential of hAESCs (Bejaoui et al., 2021; Ferdousi et al., 2021; Takahashi et al., 2021). We have also reported that isorhamnetin may have the potential to improve anti-fibrotic effects of hAESCs (Aonuma et al., 2020). Additionally, we showed that an iridoid glucoside verbenalin may enhance therapeutic potential of hAESCs for AD through targeting multiple pathologies simultaneously, including lysosomal dysfunction, pathological angiogenesis, neurometabolic aging, pathological protein aggregation, and circadian rhythms (Ferdousi et al., 2020). A recent interesting study reported that a combination of oral administration of lycopene, a carotenoid hydrocarbon found in bright red fruits and vegetables, and hAESCs transplantation could significantly ameliorate cognitive function in an *in vivo* AD model compared to a single treatment of lycopene and hAESC (Xu et al., 2021). Additionally, combination treatment of lycopene and hAESC also improved immunosuppressive activities in chroid plexus of AD rats. In Table 1, biological functions of different compounds in hAESCs are listed. We envision the emerging combination of naturally occurring compounds and hAESCs will offer additional opportunities for successful clinical translation of hAESC.

**TABLE 1:** Biological functions of natural compounds in hAESCs.

Compound	Methodology	Differentiation direction	Biological functions (Enriched Gene Ontology and KEGG pathway)	References
Cyanidin 3-glucoside	Whole genome transcriptome analysis on day 7 cell treatment	Towards adipocyte differentiation	Inhibits cell cycle-related gene expression and induces positive regulation of fibroblast growth factor receptor signaling pathway (GO:0045743), response to muscle activity (GO:0014850)	Takahashi et al. (2021)
Ethanol extract of *Aurantiochytrium*-derived squalene	Whole genome transcriptome analysis on day 7 cell treatment	Towards neuronal differentiation	Induces positive regulation of neuron differentiation (GO:0045666), positive regulation of MAPK cascade (GO:0043410), fibroblast growth factor receptor signaling pathway (GO:0008543), regulation of lipid biosynthetic process (GO:0046890), cellular response to oxidative process	Ferdousi et al. (2021)
Isorhamnetin	Whole genome transcriptome analysis on day 10 cell treatment, functional analysis	Towards hepatic-lineage specific differentiation	Positive regulation of canonical Wnt signaling pathway (GO:0090263) and TGFb receptor signaling pathway (GO:0007179), cell-matrix adhesion	Uchida et al. (2020)
			(GO:0007160), extracellular matrix organization (GO:0030198)	
Rosmarinic acid	Whole genome transcriptome analysis on day 7 cell treatment	Towards neuronal differentiation	neurogenesis (GO: 0022008), and neuron	Ferdousi et al. (2019)
			differentiation (GO: 0030182), Chemical synaptic transmission (GO:0007268)	
3,4,5-tri-O-caffeoylquinic acid (TCQA)	Whole genome transcriptome analysis on day 7 cell treatment	Towards neuronal and pigment cell differentiation	pigment cell differentiation (GO: 0050931), neurogenesis (GO: 0022008), MAPK cascade, downregulates the expressions of inflammatory cytokines, inhibits cell cycle progression	Bejaoui et al. (2021)
Verbenalin	Whole genome transcriptome analysis on day 7 cell treatment, functional analysis	—	positive regulation of dendrite development (GO: 1900006), negative regulation of type 2 immune response (GO: 0002829), ErbB and MAPK signaling pathways	Ferdousi et al. (2020)
Lycopene	Combination treatment with lycopene and hAESCs in AD rat model	—	Ameliorates Aβ-induced neuroinflammation *in vivo*	Xu et al. (2021)

### hAESCs as a Drug Screening Tool for Natural Compounds

Human PSCs, including both ESCs and iPSCs, have been used extensively as physiologically relevant *in vitro* human models in high-throughput drug screening, from target identification to preclinical compound evaluation. Stem cell-based methods reduce the timelines and attrition rate of new therapeutics (McNeish, 2004; Ebert and Svendsen, 2010; Laustriat et al., 2010; Grskovic et al., 2011; Rubin and Haston, 2011; Engle and Puppala, 2013). However, limited cell resources, invasive extraction procedures, expensive cell reprogramming and maintenance procedures, and ethical constraints are the main challenges for the large-scale use of ESCs and iPSCs for drug screening and toxicity testing.

On the other hand, a huge number of small molecules derived from or based on natural compounds become available for drug screening and biological investigations each year. However, despite substantial technological advances, the rate of new medicine discovery is exceptionally low. Indeed, drug discovery is greatly hampered by the gap between the validation of the compound and its successful clinical application. The unpredictability of the currently used *in vitro* cellular models, where the crucial elements of drug-biology interaction are lost, and the complexity of the *in vivo* microenvironment are behind the translational inefficiency of new target compounds.

**FIGURE 2:**
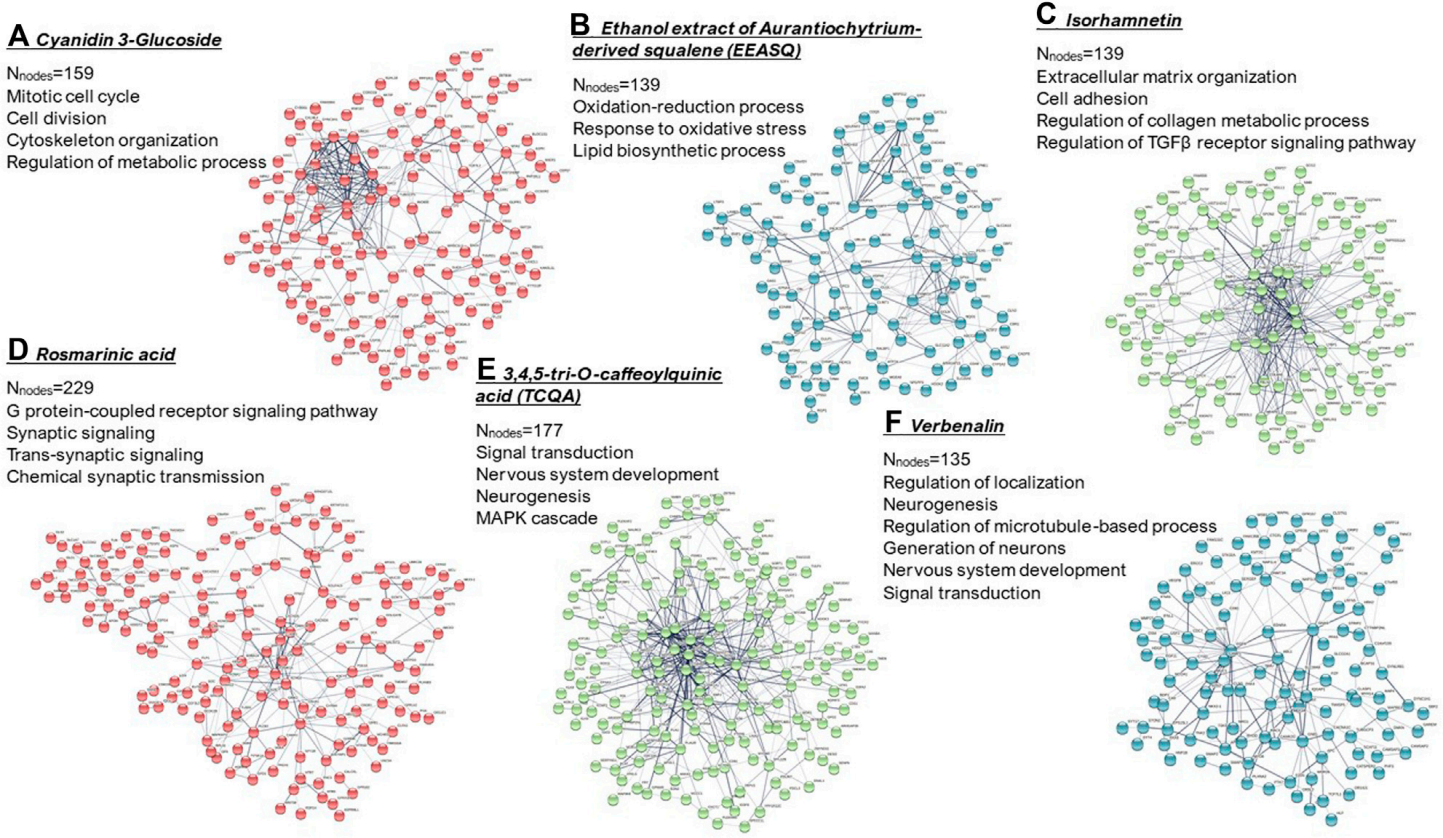
k-means clustering and PPI network of the differentially expressed genes (computed using STRING; https://string-db.org/). Significantly enriched gene ontologies (biological processes) in each network are presented. N_nodes_ = number of nodes. **(A)** Cyanidin 3-glucoside; **(B)** ethanol extract of Aurantiochytrium-derived squalene (EEASQ); **(C)** Isorhamnetin; **(D)** Rosmarinic acid; **(E)** 3,4,5-tri-O-caffeoylquinic acid (TCQA); **(F)** Verbenalin. Cells were treated with compounds for 7–10 days and RNAs were isolated from the control and treated hAESCs for microarray experiments using the Affymetrix’s GeneAtlas^®^ System (Affymetrix Inc., Santa Clara, CA, USA, human genome array strips; HG-U219). Genes with a linear fold change >1.1 (verbenalin), 1.2 (RA, TCQA and EEASQ), and 2 (ISO, CY3G) and a *p*-value < 0.05 (one-way between-subjects ANOVA) were considered as differentially expressed genes.

In this regard, hAESCs and other perinatal stem cells, which are derived from biological waste products, may offer promising cell sources in drug screening and toxicity testing efforts. In Figure 2, we have shown important biological functions of different natural compounds observed in hAESCs (please refer to Supplementary Figure file for details). In hAESCs, isorhamnetin showed anti-fibrotic potential, which was then validated in the cardiac fibrosis *in vivo* model (Aonuma et al., 2020). The observed neuroprotective potential of microalgae-derived squalene (Ferdousi et al., 2021) has also been validated in aging model mice (Sasaki et al., 2019b; Sasaki et al., 2020). Similarly, the chemical synaptic transmission activity of RA was observed in depression model mice (Sasaki et al., 2013; Kondo et al., 2015), and the neurogenesis-regulating effect of TCQA was confirmed in aging mice (Sasaki et al., 2019a). Our observations strongly suggest that hAESCs would provide a promising platform to perform initial functionality screening of natural compounds.

## Discussion

Biologically active compounds have been incorporated into stem cell research to maintain stem cell proliferation or to facilitate controlled differentiation into more defined tissues (Udalamaththa et al., 2016; Udagama and Udalamaththa, 2018; Saud et al., 2019). Our previous studies have suggested the potential of natural compounds in optimizing the microenvironment and regulating the early biological events to induce directed differentiation of hAESCs. Although hAESCs have already been studied extensively for their therapeutic potential (Toda et al., 2007), we anticipate that the emerging combination of natural compounds and hAESCs would lead to a stable molecular signature, enhanced proliferation capacity, and improved therapeutic efficacy.

One of the major challenges in hAESCs research is the heterogeneity in primary amnion-derived epithelial cell populations based on their cell surface profiling (Centurione et al., 2018; Ghamari et al., 2020). For example, studies showed that NANOG is expressed in only 1–3% of hAESCs, about 50% of term hAESCs express SSEA-4, and co-expression of SSEA-4, TRA1-60, and TRA1-81 is found in 4% of amniotic epithelial cells (Miki et al., 2005; Miki and Strom, 2006; Miki et al., 2007; Bryzek et al., 2013). Additionally, hAESCs derived from different areas of amniotic membrane exhibited different pluripotent surface markers expression and proliferative ability (Centurione et al., 2018). However, several studies have proposed better controllable approaches for generating hAESCs homogeneous enough for biological and clinical application (Miki et al., 2010; Murphy et al., 2010; Zhou et al., 2013; Gramignoli et al., 2016; Gottipamula and Sridhar, 2018; Yang et al., 2018). Another study showed that expansion of hAESCs in 3D culture system and subsequent isolation from the adherent subpopulations may enhance the stemness properties of hAESCs (Furuya et al., 2019).

From one human term amniotic membrane, nearly 200 million hAESCs can be harvested, allowing sufficient cell supply for large-scale use in academic research, pharmaceutical industry, and clinical application. For our studies on natural compound-treated hAESCs, we received the cells from ‘The Tsukuba Human Tissue Biobank Center (THB)’ established at the University of Tsukuba Hospital in 2013 (Takeuchi et al., 2016). The hAESCs were isolated from the mothers’ donated placenta who underwent cesarean section. Biobanking of perinatal stem cells began over three decades ago with the establishment of umbilical cord blood biobank. However, as the field of perinatal cells and regenerative medicine is progressing rapidly, biobanking of other types of perinatal stem cells, including hAESCs, will be an integral part of successful cell-based therapy.

Recent advances in genome-wide expression profiling, single-cell multi-omics analysis followed by machine learning-based analyses permit systematic approaches to the biological discovery of regulatory mechanisms and biochemical pathways (Chavan et al., 2006; Kumar et al., 2012). They have indeed provided certain unique opportunities for widening the application of hAESC research platform.

In the future, integrating natural compounds to hAESCs to establish an optimal culture condition, to achieve appropriate preconditioning for enhancing the therapeutic potential would be new opportunities for further investigation.

## Data Availability

Publicly available datasets were analyzed in this study. This data can be found here: Microarray data are deposited in the Gene Expression Omnibus (GEO) under accession numbers GSE148776 (CY3G), GSE188411 (EEASQ), GSE148777 (Isorhamnetin), GSE133277 (Rosmarinic acid), GSE153617 (TCQA), and GSE137061 (Verbenalin).
