# Supplementary material for: Regulating Early Biological Events in Human Amniotic Epithelial Stem Cells Using Natural Bioactive Compounds: Extendable Multidirectional Research Avenues
Source: Front Cell Dev Biol. 2022 Apr 1;10:865810. doi: 10.3389/fcell.2022.865810 (PMC9011193; doi:10.3389/fcell.2022.865810)
Supplement: Supplementary file 1 [file DataSheet1.PDF]

Supplementary Material

Regulating Early Biological Events in Human Amniotic Epithelial Stem Cells Using Natural Bioactive Compounds: Extendable Multidirectional Research Avenues

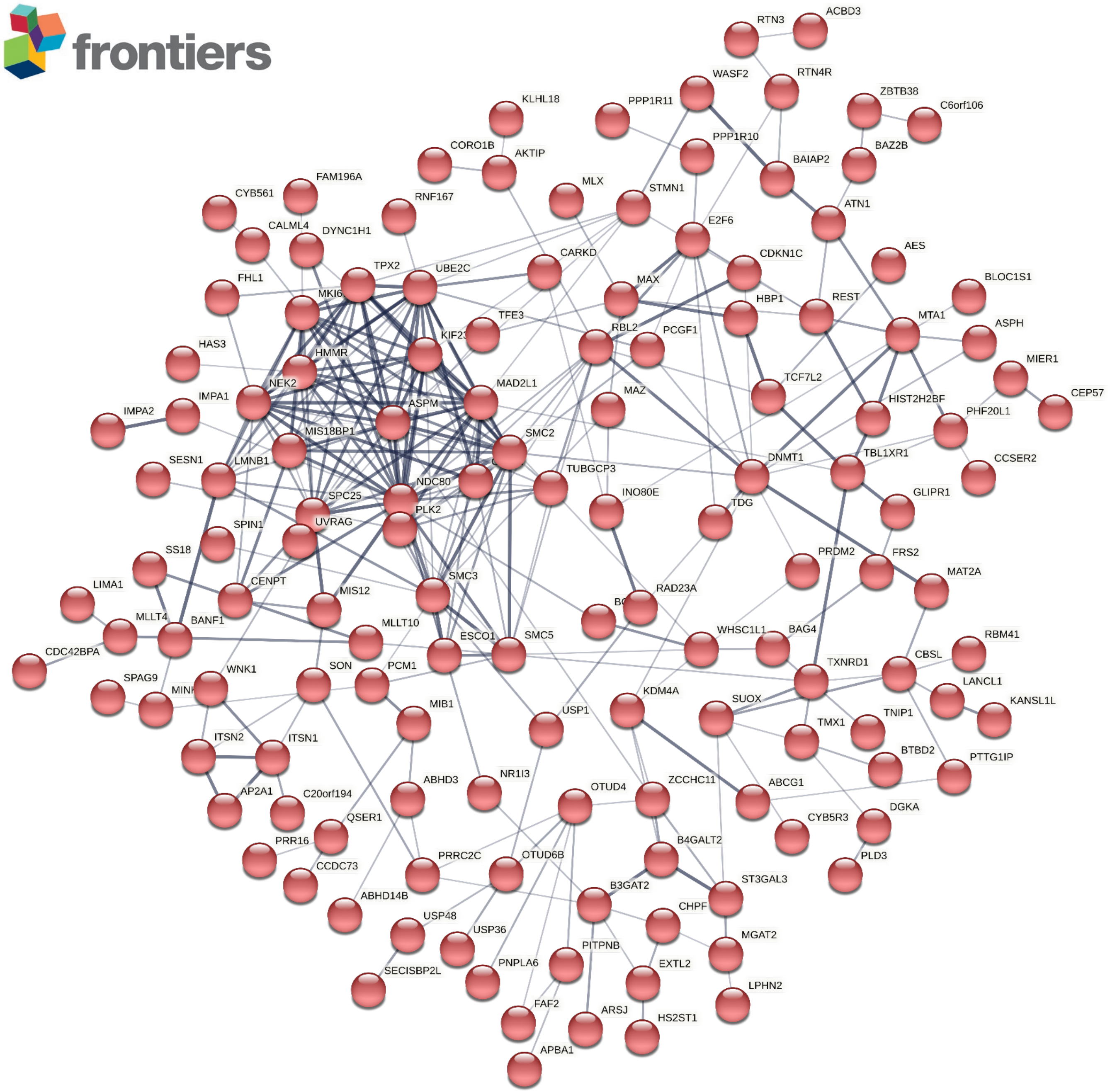

Cyanidin 3-Glucoside

number of nodes: 159
number of edges: 302
average node degree: 3.8
avg. local clustering coefficient: 0.398
expected number of edges: 90
PPI enrichment p-value: < 1.0e-16

Biological Process term ID	Term description	Observed gene count	False Discovery Rate
GO:0007049	Cell cycle	34	3.36E-05
GO:0000278	Mitotic cell cycle	23	5.15E-05
GO:0051301	Cell division	19	7.07E-05
GO:0007010	Cytoskeleton organization	25	0.0047
GO:0019222	Regulation of metabolic process	83	0.009

Supplementary Figure: k-means clustering and PPI network of the differentially expressed genes by cyanidin 3-glucoside (computed using STRING; https://string-db.org/). Significantly enriched gene ontologies (biological processes) in the network are presented.

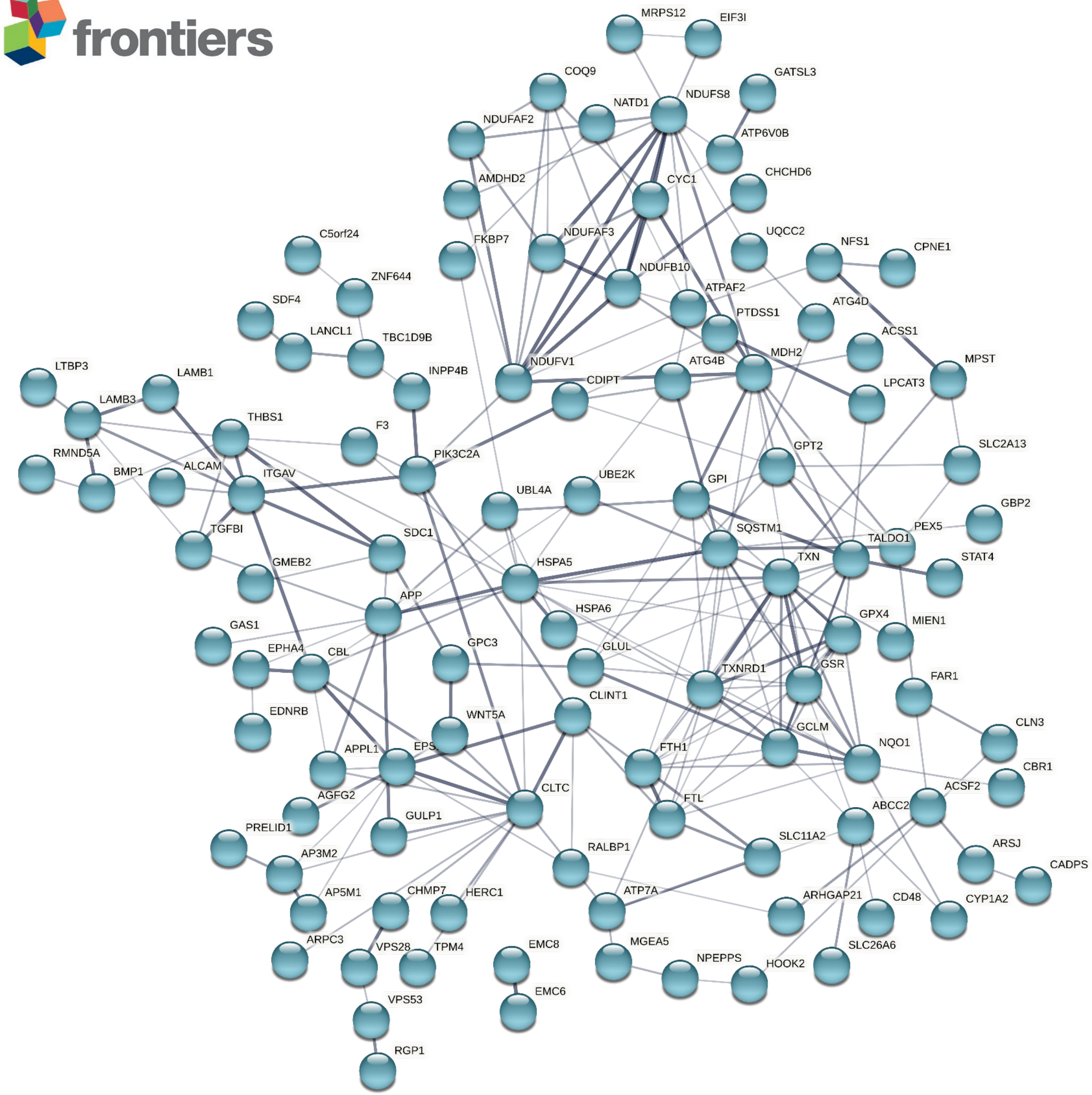

Ethanol extract of Aurantiochytrium-derived squalene (EEASQ)

number of nodes: 139
number of edges: 231
average node degree: 3.32
avg. local clustering coefficient: 0.361
expected number of edges: 84
PPI enrichment p-value: < 1.0e-16

Biological Process term ID	Term description	Observed gene count	False Discovery Rate
GO:0006091	Generation of precursor metabolites and energy	14	0.014
GO:0019725	Cellular homeostasis	20	0.0232
GO:0055114	Oxidation-reduction process	20	0.0232
GO:0006979	Response to oxidative stress	12	0.03
GO:0045333	Cellular respiration	8	0.03
GO:0008610	Lipid biosynthetic process	14	0.0451

Supplementary Figure: k-means clustering and PPI network of the differentially expressed genes by ethanol extract of *Aurantiochytrium*-derived squalene (EEASQ) (computed using STRING; https://string-db.org/). Significantly enriched gene ontologies (biological processes) in the network are presented.

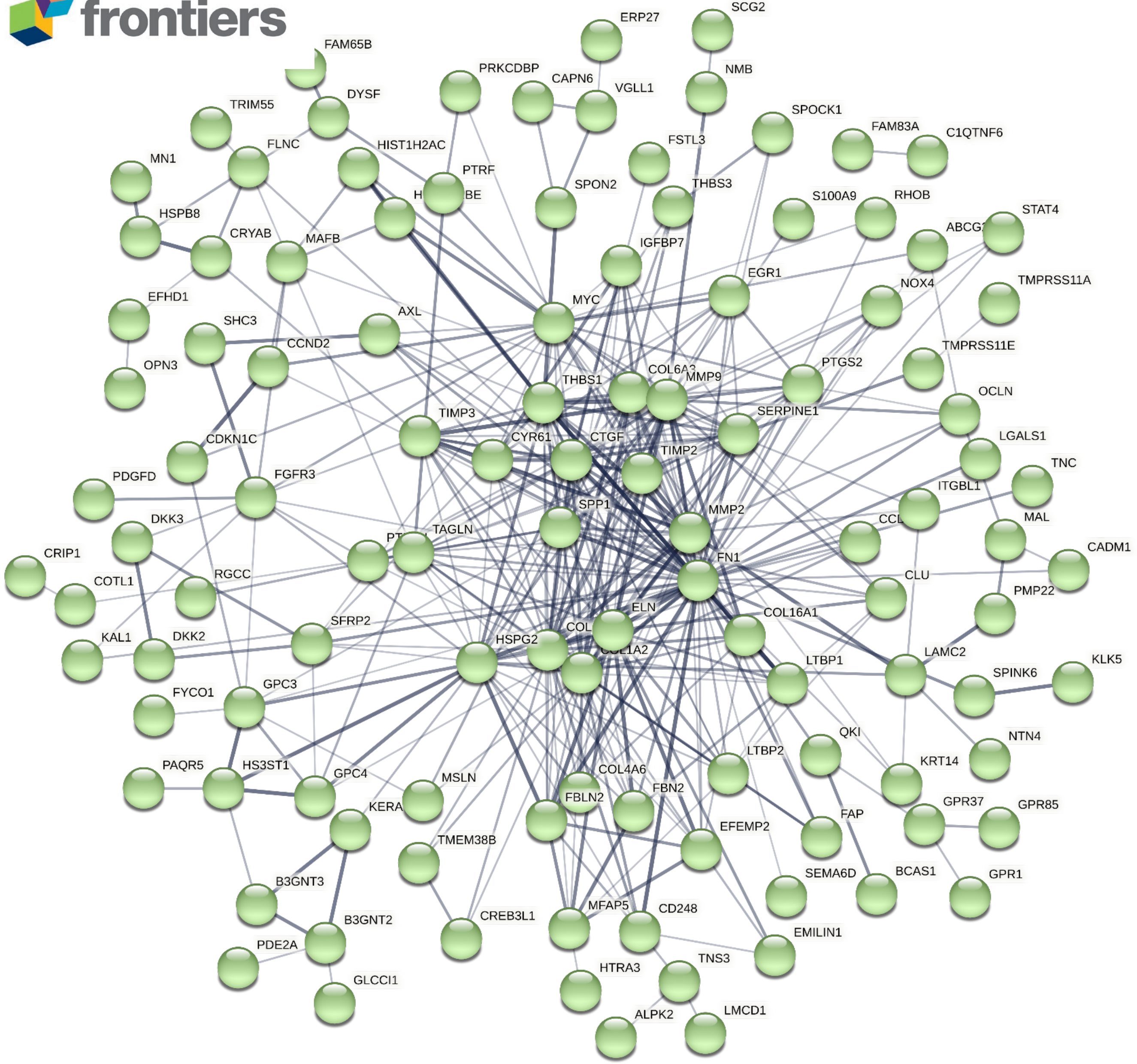

Isorhamnetin

number of nodes: 139
number of edges: 372
average node degree: 5.35
avg. local clustering coefficient: 0.488
expected number of edges: 84
PPI enrichment p-value: < 1.0e-16

Biological Process term ID	Term description	Observed gene count	False Discovery Rate
GO:0030198	Extracellular matrix organization	29	4.22E-18
GO:0007155	Cell adhesion	31	1.65E-09
GO:0090287	Regulation of cellular response to growth factor stimulus	12	0.00047
GO:0010712	Regulation of collagen metabolic process	5	0.0039
GO:0017015	Regulation of transforming growth factor beta receptor signaling pathway	7	0.0057

Supplementary Figure: k-means clustering and PPI network of the differentially expressed genes by isorhamnetin (computed using STRING; https://string-db.org/). Significantly enriched gene ontologies (biological processes) in the network are presented.

frontiers

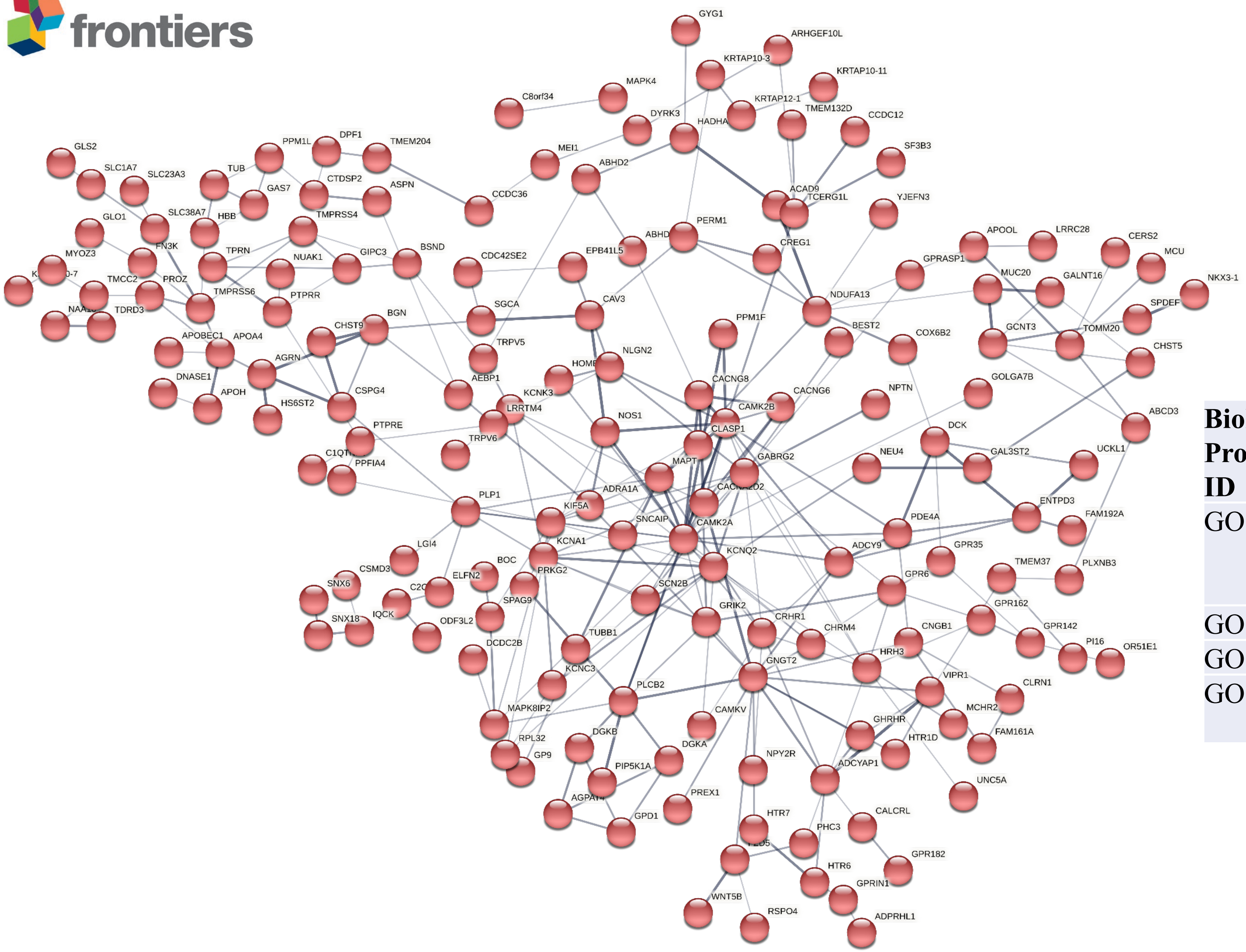

Rosmarinic Acid

number of nodes: 229
number of edges: 274
average node degree: 2.39
avg. local clustering coefficient: 0.327
expected number of edges: 94
PPI enrichment p-value: < 1.0e-16

Biological Process term ID	Term description	Observed gene count	False Discovery Rate
GO:0007186	G protein-coupled receptor signaling pathway	39	0.00058
GO:0099536	Synaptic signaling	20	0.0082
GO:0099537	Trans-synaptic signaling	19	0.0092
GO:0007268	Chemical synaptic transmission	17	0.0471

Supplementary Figure: k-means clustering and PPI network of the differentially expressed genes by rosmarinic acid (computed using STRING; https://string-db.org/). Significantly enriched gene ontologies (biological processes) in the network are presented.

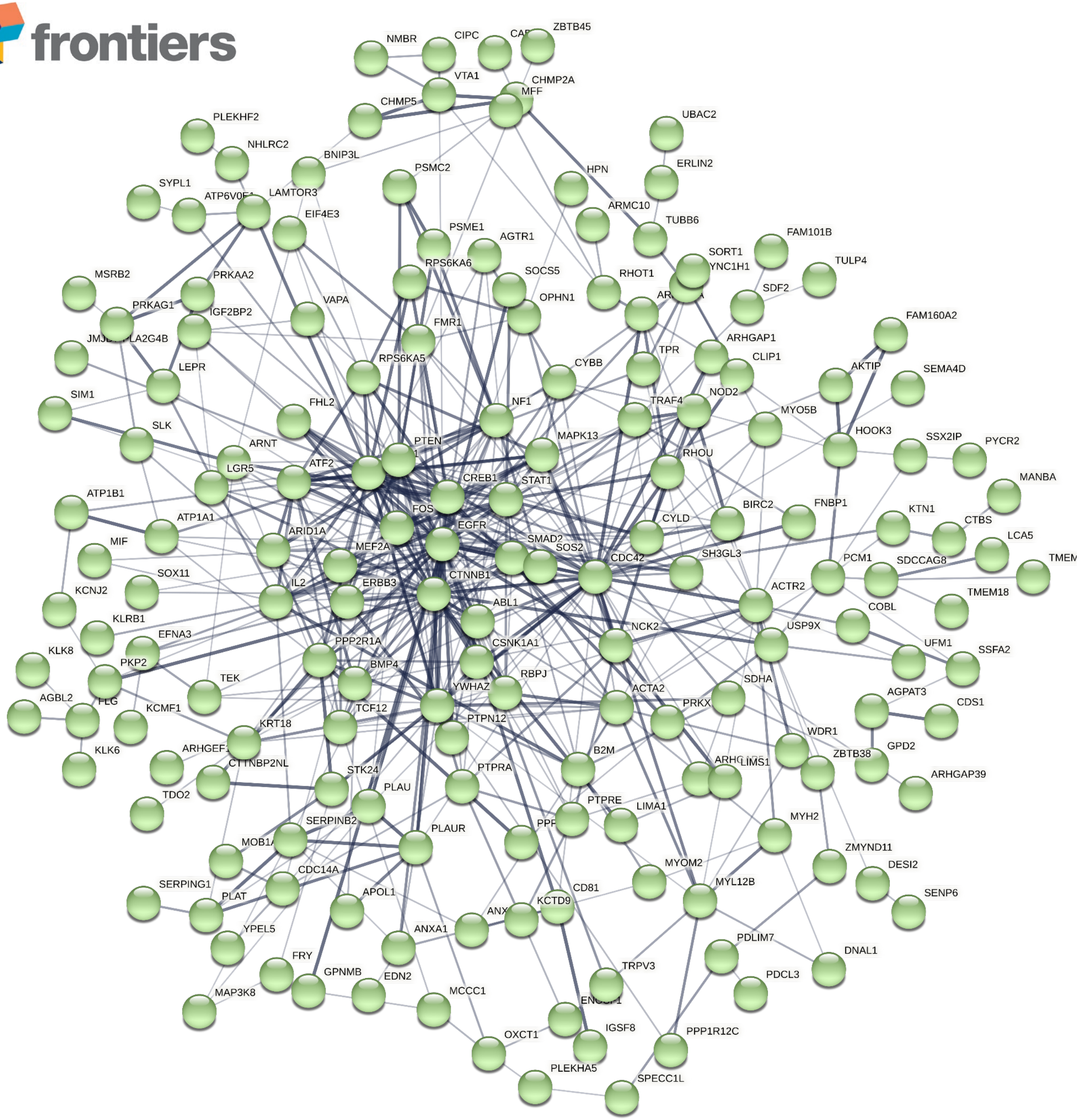

3,4,5-tri-O-caffeoylquinic acid (TCQA)

number of nodes: 177
number of edges: 498
average node degree: 5.63
avg. local clustering coefficient: 0.443
expected number of edges: 224
PPI enrichment p-value: < 1.0e-16

Biological Process term ID	Term description	Observed gene count	False Discovery Rate
GO:0007165	Signal transduction	90	9.25E-10
GO:0007154	Cell communication	94	1.73E-09
GO:0007399	Nervous system development	51	1.40E-06
GO:0022008	Neurogenesis	41	1.62E-06
GO:0048699	Generation of neurons	38	6.81E-06
GO:0030155	Regulation of cell adhesion	24	1.42E-05
GO:0007346	Regulation of mitotic cell cycle	23	1.98E-05
GO:0071345	Cellular response to cytokine stimulus	28	3.83E-05
GO:0000165	MAPK cascade	15	0.00025

Supplementary Figure: k-means clustering and PPI network of the differentially expressed genes by 3,4,5-tri-O-caffeoylquinic acid (TCQA) (computed using STRING; https://string-db.org/). Significantly enriched gene ontologies (biological processes) in the network are presented.

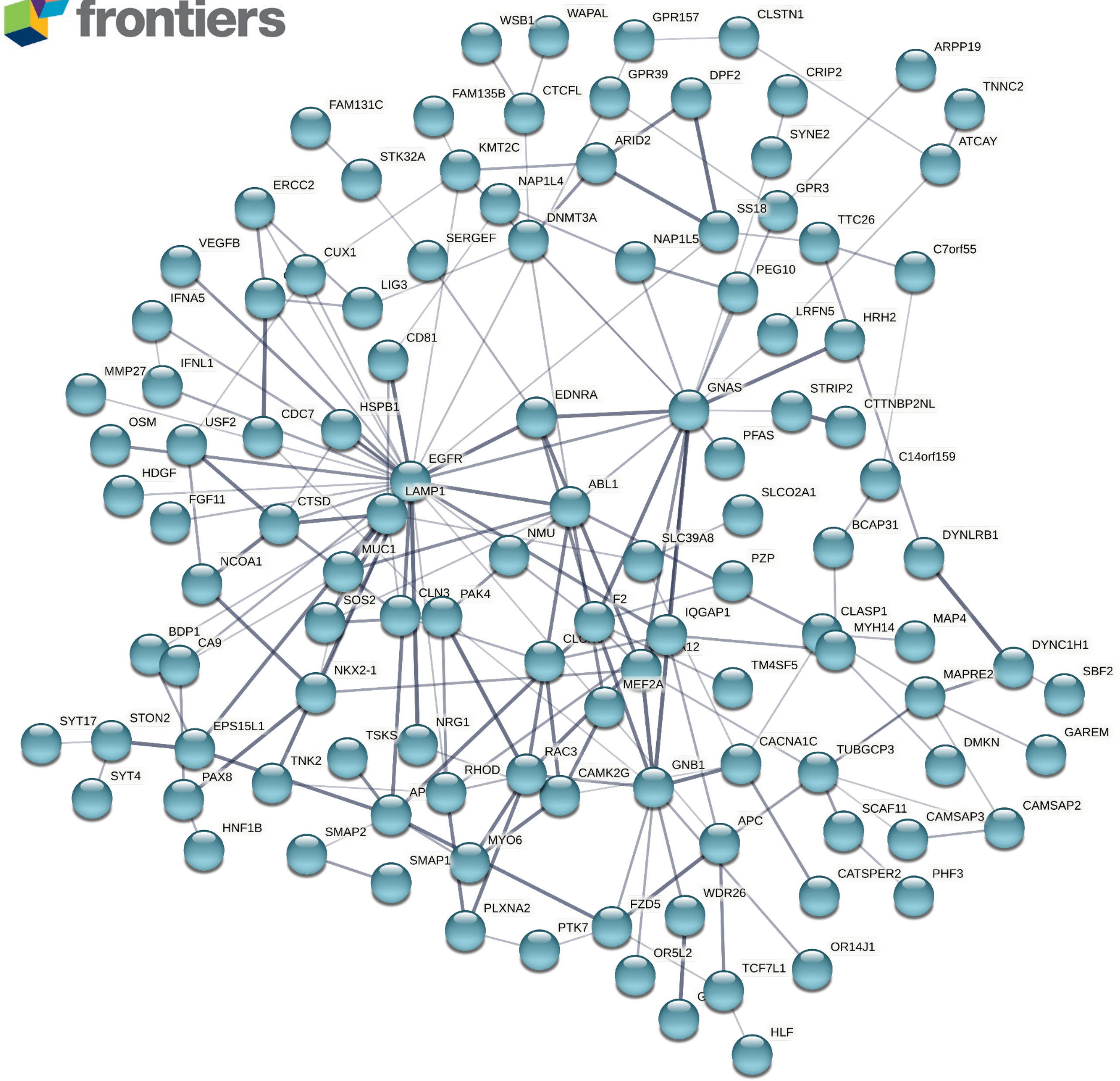

Verbenalin

number of nodes: 135

number of edges: 184

average node degree: 2.73

avg. local clustering coefficient: 0.371

expected number of edges: 76

PPI enrichment p-value: < 1.0e-16

Biological Process term ID	Term description	Observed gene count	False Discovery Rate
GO:0032879	Regulation of localization	47	2.12E-05
GO:0051128	Regulation of cellular component organization	37	0.0036
GO:0022008	Neurogenesis	28	0.009
GO:0032886	Regulation of microtubule-based process	10	0.0095
GO:0048699	Generation of neurons	26	0.0164
GO:0007399	Nervous system development	34	0.0171
GO:0007165	Signal transduction	55	0.0245

Supplementary Figure: k-means clustering and PPI network of the differentially expressed genes by verbenalin (computed using STRING; https://string-db.org/). Significantly enriched gene ontologies (biological processes) in the network are presented.
